# Conjoint application of nano-urea with conventional fertilizers: An energy efficient and environmentally robust approach for sustainable crop production

**DOI:** 10.1371/journal.pone.0284009

**Published:** 2023-07-05

**Authors:** Pravin Kumar Upadhyay, Abir Dey, Vinod Kumar Singh, Brahma Swaroop Dwivedi, Tarunendu Singh, Rajanna G. A., Subhash Babu, Sanjay Singh Rathore, Rajiv Kumar Singh, Kapila Shekhawat, Meenakshi Rangot, Pradeep Kumar, Dhinu Yadav, Devendra Pratap Singh, Debarshi Dasgupta, Gaurav Shukla

**Affiliations:** 1 Division of Agronomy, ICAR-Indian Agricultural Research Institute, New Delhi, India; 2 Division of Soil Science and Agricultural Chemistry, ICAR-Indian Agricultural Research Institute, New Delhi, India; 3 ICAR-Central Research Institute for Dryland Agriculture, Hyderabad, India; 4 Agricultural Scientists Recruitment Board, New Delhi, India; 5 Indian Farmers Fertiliser Cooperative Limited, New Delhi, India; 6 ICAR- Directorate of Groundnut Research, Regional Station- Ananthapur, Ananthapur, India; 7 GD Goenka University, Sohna, Haryana, India; 8 Nano Fertilizer Plant, Indian Farmers Fertiliser Cooperative Limited, Prayagraj, India; 9 Chandra Shekhar Azad University of Agriculture & Technology, Kanpur, India; 10 North Dakota State University, Fargo, ND, United States of America; DRMR: Directorate of Rapeseed-Mustard Research, INDIA

## Abstract

One of the biggest challenges to be addressed in world agriculture is low nitrogen (N) use efficiency (<40%). To address this issue, researchers have repeatedly underlined the need for greater emphasis on the development and promotion of energy efficient, and environmentally sound novel fertilizers, in addition to improved agronomic management to augment nutrient use efficiency for restoring soil fertility and increasing farm profit. Hence, a fixed plot field experiment was conducted to assess the economic and environmental competency of conventional fertilizers with and without nano-urea (novel fertilizer) in two predominant cropping systems *viz*., maize-wheat and pearl millet-mustard under semi-arid regions of India. Result indicates that the supply of 75% recommended N with conventional fertilizer along with nano-urea spray (N_75_PK+nano-urea) reduced the energy requirement by ~8–11% and increased energy use efficiency by ~6–9% over 100% nitrogen through prilled urea fertilizer (business as usual). Furthermore, the application of N_75_PK+ nano-urea exhibited ~14% higher economic yields in all the crops compared with N_50_PK+ nano-urea. Application of N_75_PK+nano-urea registered comparable soil N and dehydrogenase activities (35.8 μg TPF g^-1^ 24 hrs^-1^ across all crops) over the conventional fertilization (N_100_PK). This indicates that application of foliar spray of nano-urea with 75% N is a soil supportive production approach. More interestingly, two foliar sprays of nano-urea curtailed nitrogen load by 25% without any yield penalty, besides reducing the greenhouse gases (GHG) emission from 164.2 to 416.5 kg CO_2_-eq ha^-1^ under different crops. Therefore, the application of nano-urea along with 75% N through prilled urea is an energy efficient, environmentally robust and economically feasible nutrient management approach for sustainable crop production.

## 1. Introduction

Conventional chemical fertilizers are widely used across the globe for achieving maximum yield in agricultural systems to meet the demand of food for ever-growing population. The post green revolution era witnessed an excess and unchecked use of chemical fertilizers across the globe. With an increasing world population [1] and diminishing arable land [2], it is becoming necessary to employ larger quantities of chemical fertilizers [3], especially nitrogen (N) to meet the global food demands [4]. In 2019–20, worldwide total demand of N was 107.4 million tonnes and out of it 76.5% was supplied through urea [5]. The worldwide demand of urea is increasing day by day, which has increased from 147.5 Mt in 2009 to 178.3 Mt in 2019 [5], indicating 20.9% rise in its demand in a decade. The major concern of global urea production and use is environmental pollution. GHG emissions (urea production + use) per kg use of urea is 5.15 kg CO_2_-eq [6]. This means globally huge emission of about 918 Mt CO_2_-eq took place from total urea production in 2019. Water and specific electricity consumption for the production of a tonne of urea is 12.8 m^3^ and 173.7 kWh, respectively [7]. Hence, a huge quantity of water (228×10^7^ m^3^) and electricity (3097×10^7^) consumption took place in 2019 due to the production of 178.3 Mt urea worldwide. Large consumption of water and electricity, and massive emission of GHGs in the urea production may have a severe harmful impact on the environment. Consequently, identifying potential points for improving efficiency of water and electricity and reducing the GHGs emission under urea production system may be one way, but reducing total urea demand over the globe by replacing urea with energy efficient novel fertilizers [8] would be a more viable approach.

Under field condition, N use efficiencies of conventional fertilizers rarely exceed 30–35% [9]. Therefore, application of these fertilizers in excessive amount for supplying nutrient demand of the crops has environmental and ecological consequences [10]. Nutrient losses from agricultural soils in the form of leaching (NO_3_¯) or gaseous emissions (NH_3_ and N_2_O) leads to environmental pollution [11]. Scientists, policymakers, industrialists and farmers are equally concerned over these facts [12], and are actively looking for some alternate novel nutrient sources. For the last two decades, development of nano-fertilizers has been researched upon by various researchers, organizations and institutes [13]. The novel nano-fertilizers are believed to have the potential for a paradigm shift in agriculture [8]. However, so far there is hardly any systematic study that has shown the effect of nano fertilizers or integration of nano-fertilizers with conventional fertilizers on productivity and profitability of crops under field conditions [14, 15]. A very few examples are being seen where nanotechnology has moved from the laboratory to the field level, and even fewer where it has been deployed at the commercial scale [16]. Nano-fertilizers are nutrient carriers [15], developed using the substrates of nano-dimensions (1–100 nm) [17]. The nano-fertilizers possess more surface area [15] to volume size ratio and the feature of surface functionalization [18] along with slow or plant response-based release [19]. Therefore, nano-fertilizers can reduce nutrient losses through leaching and gaseous emissions, ensuring a sustainable production system [20]. The nano-clay-based fertilizer formulations [21] are capable of releasing N for a much longer period [22] than the conventional fertilizers. Coating of fertilizer molecules with nano-membranes also facilitates slow or controlled release fertilizers [23]. The introduction of nano-fertilizer in Indian or world agriculture can prove to be an important step towards sustainable agriculture, which promotes minimalistic use of agro-chemicals, curbing environmental pollution, promoting ecological preservation, and maintain and improving soil health with a subsequent increment in crop productivity and farmers’ income.

Recently, the Indian Farmers Fertiliser Cooperative (IFFCO) invented and patented (Indian patent application number 201921053828, and application number 201921044499) a nano-fertilizer, viz. nano-urea for use as an alternative to commercial urea. The fertilizer nano-urea has a size of particle in nanometre (nm) in one dimension (minimum 50% of the material), physical particle size ranging between 20 and 50 nm, and hydrodynamic particle size varying from 20 to 80 nm [24]. Nano-urea contains 4% N, has a shelf-life of about 2 years, and has a zeta potential > 30 [24]. It contains functional nutrients derived primarily from urea which are treated with non-ionic surfactants and further stabilised in polymer matrices to produce nano clusters of less than 100 nm size. The developed nano-fertilizer was tested in laboratory conditions, and small-scale pot studies to check its effectiveness [19, 25]. The efficacy of IFFCO invented nano-urea was tested based on multi-location (11,000 locations) and multi-crop (94 crops) trials in different crop seasons, both by the researcher’s and progressive farmers’ in India. It was found that the application of nano-urea enhanced yields in wheat [26–28] maize [29] and Indian mustard [30–32] across the locations.

The initial studies indicated a possibility of curtailing fertilizer doses with subsequent application of nano-urea. Hence, there may be possibility about use of nano-urea can reduce other forms of N fertilization application and also it can positively influence soil health thereby it might pave the way for widespread adoption of nano-fertilizers across the globe and curtail urea requirement at the same time. Therefore, a full-fledged experimental trial was conducted at ICAR-Indian Agricultural Research Institute, New Delhi (India) to assess the effectiveness of nano-urea with the objectives: 1) to assess the effect of nano-urea on crop productivity, profitability, energetics and N uptake under maize-wheat and pearl millet-mustard systems; and 2) to study the effect of nano-urea on mineral N and enzymatic activities.

## 2. Materials and methods

### 2.1 Site description

Field experiments were conducted at the research farm of ICAR-Indian Agricultural Research Institute, New Delhi (N 28.38.0838 and E 077.09.1441). The sandy loam soil of the experimental site was mildly alkaline (pH 8.22) and non-saline (EC 0.24 dS m^-1^). The plough layer (0–15 cm) soil contained 0.58% organic C, 272 kg ha^-1^ available N, 22.3 kg ha^-1^ available P, and 311 kg ha^-1^ available K. DTPA-extractable Zn, Fe, Mn and Cu contents in the soil were 0.84 mg kg^-1^, 4.72 mg kg^-1^, 19.9 mg kg^-1^ and 1.91 mg kg^-1^, respectively.

### 2.2 Experimental details

The field experiments under maize-wheat and pearl millet-mustard systems were conducted with 8 treatments comprising of 4 rates of N (0, 50, 75 and 100% of recommended N) with and without nano-urea in a randomized complete block design (RCBD) with 3 replications. Fertilizer P and K were applied uniformly at recommended rates to all plots. The complete treatment details are listed in Table 1. The maize, wheat, pearl millet and mustard crops were grown in respective cropping systems for two cropping cycles. The crop management details are given in the Table 2.

**Table 1:** Treatments details of experiments undertaken in maize-wheat and pearl millet-mustard systems.

S. No.	Treatment	Treatment details
T1	N_0_PK	Recommended P and K (no-N)
T2	N_50_PK	Recommended P, K and 50% of recommended N
T3	N_75_PK	Recommended P, K and 75% of recommended N
T4	N_100_PK	Recommended P, K and 100% of recommended N
T5	N_0_PK + nano-urea	Recommended P and K (no-N) + 2 nano-urea sprays
T6	N_50_PK+ nano- urea	Recommended P, K and 50% of recommended N + 2 nano-urea sprays
T7	N_75_PK+ nano- urea	Recommended P, K and 75% of recommended N + 2 nano-urea sprays
T8	N_100_PK+ nano- urea	Recommended P, K and 100% of recommended N + 2 nano-urea sprays

*Recommended fertilizer doses were 150 kg N ha^-1^, 75 kg P_2_O_5_ ha^-1^, 75 kg K_2_O ha^-1^ for maize; 60 kg N ha^-1^, 60 kg P_2_O_5_ ha^-1^, 30 kg K_2_O ha^-1^ for pearl millet; 80 kg N ha^-1^, 40 kg P_2_O_5_ ha^-1^, 30 kg K_2_O ha^-1^ for mustard and 120 kg N ha^-1^, 60 kg P_2_O_5_ ha^-1^, 60 kg K_2_O ha^-1^ for wheat crop.

**Table 2:** Agronomic package followed under different test crops.

Operation	Maize	Wheat	Pearl millet	Mustard
Tillage	Two ploughing with cultivator, Double discing followed by planking	Two ploughing with cultivator, Double discing followed by planking	One ploughing with cultivator, Double discing followed by planking	One ploughing with cultivator, Double discing followed by planking
Seed treatment	Thiram @2 g per kg seed	Thiram @2 g per kg seed	Thiram @2 g per kg seed	Thiram @2 g per kg seed
Variety/Hybrid	PJHM 1	HD 3086	Pioneer hybrid bajra 86M90	PM 28
Seed rate	22 kg ha^-1^	100 kg ha^-1^	5 kg ha^-1^	5 kg ha^-1^
Weed management	Pre-emergence application of Pendimethaline @ 1 l a.i. ha^-1^ + one hand weeding 22 Days after sowing	Pre-emergence application of Pendimethaline @ 1 l a.i. ha^-1^ + 75% Sulfosulfuron & 5% WG Metsulfuron@40 g a.i. ha^-1^	Pre-emergence application of Pendimethaline @ 1 l a.i. ha^-1^ + one hand weeding	Pre-emergence application of Pendimethaline @ 1 l a.i. ha^-1^ + one hand weeding
Insecticide	Emamectin benzoate 5 SG @0.4ml l^-1^	-	-	-
Fungicide	-	-	-	4% w/w metalaxyl-M and 64% w/w mancozeb @ 2.5 kg ha^-1^
Harvesting	At physiological maturity	At physiological maturity	At physiological maturity	At physiological maturity

### 2.3 Nutrient management

The recommended fertilizer doses in terms of N, P_2_O_5_ and K_2_O were 150, 75 and 75 kg ha^-1^ respectively for maize crop. In wheat, N, P_2_O_5_ and K_2_O were applied @ 120, 60 and 60 kg ha^-1^, respectively. In pearl millet, N, P_2_O_5_ and K_2_O were applied @ 60, 60 and 30 kg ha^-1^, respectively. In mustard, N, P_2_O_5_ and K_2_O were applied @ 80, 40 and 30 kg ha^-1^, respectively. The source of fertilizers were urea, single superphosphate and muriate of potash to supply N, P and K, respectively. In mustard and pearl millet, 50% of N (as per treatment) and 100% of recommended P and K were applied as basal, and the remaining 50% N was top-dressed in one split. In wheat and maize, 50% of N (as per treatments) and 100% of P and K fertilizers were applied as basal, and the remaining N was top-dressed in two equal splits. The Nano- urea was sprayed to each crop twice, first at 30 days after germination and second at one week before flowering. The nano-urea was applied @ 4 mL L^-1^. Foliar sprays of nano-urea were undertaken through hand operated knapsack sprayers with flat fan nozzles for complete coverage of leaves. During *kharif* (rainy) season, whenever rain occurred within 12 hours of spray, the process was repeated. In winter, the spraying was done during evening hours, when there was no dew present on the leaves.

### 2.4 Harvesting and yield monitoring

All crops were harvested manually and aboveground biomass was removed from the plots. The grain yields of all crops were measured from 16 m^2^ net plot area and finally the yield of net plot was converted into tonne ha^-1^. The harvested produce was sun-dried, and thrashed using mechanical thrasher. The straw and grain yields were reported separately on dry-weight basis. All crop residues were removed from the field after harvest.

### 2.5 Collection and processing of soil samples

At flowering stage of each crop in both systems, representative soil samples were collected from each plot at the depth of 0–15 cm using a core sampler (with a core of 3.9 cm diameter and 179.2 cm^3^ volume). A sub-set of the fresh undisturbed moist sample was used for analysis of mineral N, microbial biomass carbon (MBC) and dehydrogenase activity (DHA). The sub-set was stored at 4°C in sealed containers. The rest of the samples were air dried, ground in a mortar and pestle, passed through a 2-mm sieve and ultimately stored for further analysis. Another set of soil samples were taken after harvesting of each crop in both systems. One sub-set of undisturbed field-moist soil samples were used for analysis of mineral N, whereas, rest of these samples were air dried and processed for further analysis.

### 2.6 Soil and plant analysis

Dehydrogenase activities (DHA) were monitored in soil at flowering stage (S1 Table in S1 File) under maize-wheat and pearl millet-mustard system. The DHA was determined in the same samples by monitoring the rate of production of tri-phenyl formazan (TPF) from tri-phenyl tetrazolium chloride (TTC) in anaerobic conditions using the method of Casida [33]. Briefly, moist soil (2 g) was treated with 2.5 ml 3% TTC–Tris buffer (pH 7.6), and then incubated in darkness for 24 h at 25°C. All results were expressed on an oven-dried (105°C for 24 hrs followed by 65°C till constant weight gained) soil weight basis.

The mineral N was extracted with 2 M KCl from undisturbed soil samples [34] collected at flowering (S1 Table in S1 File) and post-harvest stages, and estimated following steam distillation method [35]. The available Zn and Cu were extracted from processed soil samples collected at both stages using 0.005 M diethylene tri-amine penta acetic acid (DTPA), 0.1 M tri-ethanolamine (TEA) and 0.01 M calcium chloride (CaCl_2_⋅2H_2_O), buffered at pH 7.3 [36], and then determined by using atomic absorption spectrophotometer. Grain/seed and straw/stover samples were analysed for N content following the micro-Kjeldahl method as described by Jackson [37].

### 2.7 Nitrogen uptake

Nitrogen (N) uptake by grain/seed and straw/stover of different crops was calculated in kg ha^-1^ in relation to dry matter production ha^-1^ by using the following formula [38]:

$$\begin{array}{l} \text{N}\;\text{uptake}\;\text{(kg}\;{\text{ha}}^{-1}\text{)}=\\ \frac{\text{N}\;\text{content}\;(\%)\;\text{in}\;\text{grain}\;\text{or}\;\text{seed}\;\text{and}\;\text{straw}\;\text{or}\;\text{stover}\;\times \;\text{Grain}\;\text{or}\;\text{seed}\;\text{and}\;\text{straw}\;\text{or}\;\text{stover}\;\text{yield}\;(\text{kg}\;{\text{ha}}^{-1})}{\text{100}} \end{array}$$

### 2.8 Economic analysis

The economic analysis included studying the cost of cultivation, gross returns, net returns, and benefit: cost (B:C) with or without subsidy of urea in different treatments. The cost of cultivation was calculated for all treatments with the prevailing market prices of inputs and worked out by considering all the expenses incurred in the cultivation of each crop and summed up with the common costs of various operations and inputs. Gross profit was calculated by multiplying the grain and straw yield ha^-1^ with the prevailing market prices of grain and straw, respectively. Benefit-cost ratio (B:C) was calculated by dividing the net return to the cost of cultivation of the individual treatment combination.


$$\text{B}:\text{C}=\frac{\text{Gross}\;\text{return}}{\text{Cost}\;\text{of}\;\text{cultivation}}$$

### 2.9 Energy use efficiency

All inputs (fertilizers, seeds, fuel, human, agro-chemicals, implements, machine etc.) and outputs (main and by-product) were taken for energy computations. Physical unit of inputs were translated in to energy units [39] by multiplying with energy equivalents (S2 Table in S1 File) as suggested by Devasenapathy et al. [40] for the estimation of energy inputs. Similarly, by multiplying the amount of grain/seed and straw/stover yield by its corresponding energy equivalent [40] the energy output [39] was calculated. The net energy and energy use efficiency were calculated as described below.


$$\text{Net}\;\text{energy}\;(\text{MJ}\;{\text{ha}}^{-1})=\text{Energy}\;\text{output}(\text{MJ}\;{\text{ha}}^{-1})-\text{Energy}\;\text{input}\;(\text{MJ}\;{\text{ha}}^{-1})$$

$$\text{Energy}\;\text{use}\;\text{efficiency}\;(\text{per}\;\text{cent})=\frac{\text{Energy}\;\text{input}}{\text{Energy}\;\text{output}}\times 100$$

### 2.10 GHG emission

Greenhouse gas (GHG) emissions was calculated using reference values 5.15 kg CO_2_-eq kg^-1^ product (urea production + use) [6], and value for GHG emission was taken 0.248 kg CO_2_-eq L^-1^ product based on the steam and power consumption of nano-urea plant (measured).

### 2.11 Statistical analysis

**Fig 1:**
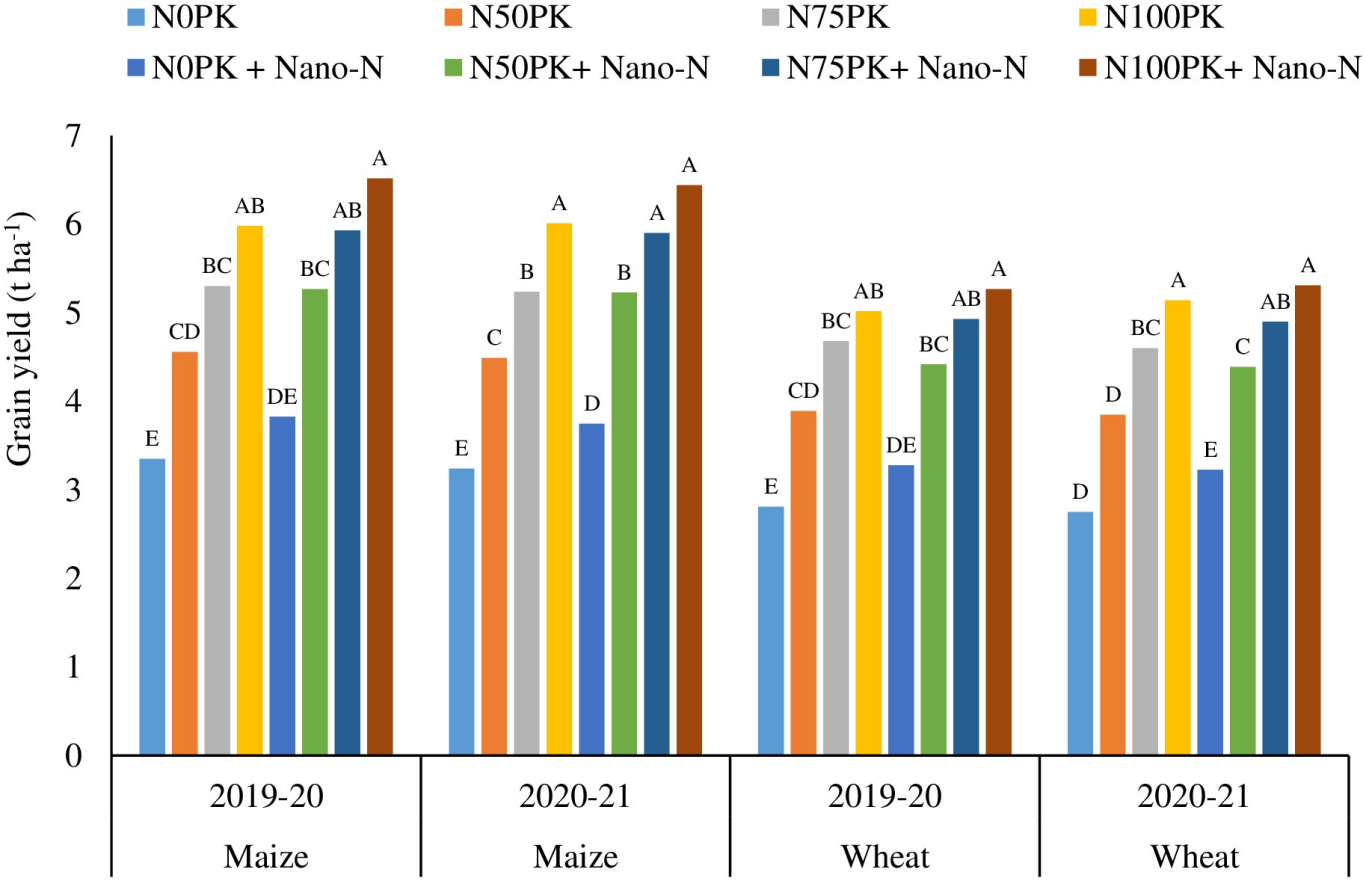
Effect of nano-urea on grain yield (t ha^-1^) of maize and wheat. *Values of means followed by different capital letter(s) based on duncan’s multiple range tests are significantly different at p ≤0.05.

**Fig 2:**
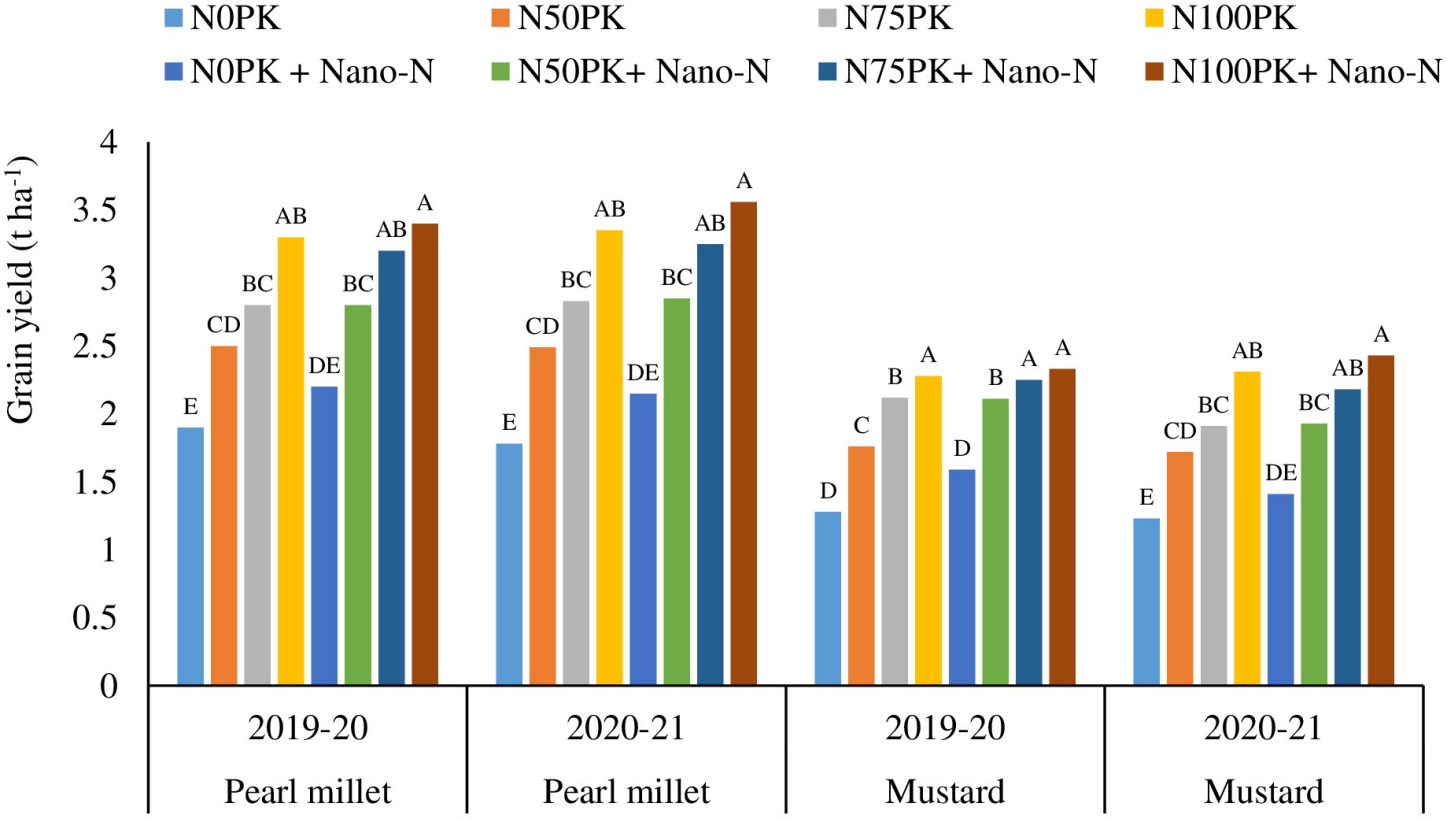
Effect of nano-urea on grain yield (t ha^-1^) of pearl millet and mustard. *Values of means followed by different capital letter(s) based on duncan’s multiple range tests are significantly different at p ≤0.05.

The data were subjected to analysis of variance (ANOVA) of a RCBD and tested at 5% level of significance (Tables 3–6, 8; S3-S6 Tables in S1 File, and Figs 1 and 2) using SAS 9.3. *Post-hoc* mean separation was done using Duncan’s Multiple Range Test. The analysis was made following fixed effect model [41]

$$y_{ij}=\mu +\tau_{i}+\beta_{j}+\epsilon_{ij}$$

$$\tau_{i}\left(i=1,2,\ldots ,8\right),\;\text{and}\;\beta_{j}\left(j=1,2,3\right)$$

where, *y*_*ij*_ is the measurement on the unit in block *j* that received treatment *i*, *μ* is the mean, *τ*_*i*_ is the *i*^*th*^ treatment effect, *β*_*j*_ is the *j*^*th*^ block effect, and *ϵ*_*ij*_ is the random error of the observation.

**Table 3:** Effect of nano-urea on mineral nitrogen (μg g^-1^ of soil) at flowering and post-harvest stages in maize-wheat system.

Treatments	Maize				Wheat			
	2020		2021		2019–20		2020–21	
	Flowering	Post-harvest	Flowering	Post-harvest	Flowering	Post-harvest	Flowering	Post-harvest
N_0_PK	19.5^D^	16.54^E^	19.1^C^	15.5^D^	20.7^D^	17.6^D^	20.8^D^	17.8^C^
N_50_PK	22.6^BCD^	20.88^D^	23.3^BC^	19.6^CD^	23.6^CD^	21.2^CD^	26.7^BC^	23.1^ABC^
N_75_PK	25.2^BC^	23.13^CD^	26.6^AB^	23.5^ABC^	25.3^BCD^	23.9^BCD^	27.9^ABC^	25.0^AB^
N_100_PK	30.7^A^	28.53^AB^	31.9^A^	28.0^AB^	30.8^AB^	28.6^AB^	30.9^AB^	27.9^A^
N_0_PK + Nano-urea	21.4^CD^	20.37^DE^	21.1^BC^	18.9^CD^	22.2^CD^	21.5^CD^	21.4^D^	19.5^C^
N_50_PK+ Nano- urea	23.3^BCD^	22.78^CD^	23.2^BC^	21.8^BC^	24.1^CD^	21.3^CD^	25.1^CD^	22.1^BC^
N_75_PK+ Nano- urea	26.9^AB^	25.03^BC^	26.4^AB^	25.1^ABC^	27.7^ABC^	25.3^ABC^	27.7^ABC^	25.1^AB^
N_100_PK+ Nano- urea	31.5^A^	30.40^A^	31.3^A^	29.7^A^	32.2^A^	30.2^A^	31.8^A^	27.6^A^

*Values of means followed by different capital letter(s) (based on Duncan’s multiple range tests) within the row are significantly different at p ≤0.05.

**Table 4:** Effect of nano-urea on mineral nitrogen (μg g^-1^ of soil) at flowering and post-harvest stages of pearl millet-mustard system.

Treatments	Pearl millet				Mustard			
	2020		2021		2019–20		2020–21	
	Flowering	Post-harvest	Flowering	Post-harvest	Flowering	Post-harvest	Flowering	Post-harvest
N_0_PK	20.8^B^	19.0^B^	20.2^B^	17.9	22.3	19.6^B^	22.3^C^	19.3^D^
N_50_PK	25.8^AB^	24.6^AB^	25.1^AB^	23.5	27.3	25.4^AB^	27.3^BC^	24.0^C^
N_75_PK	29.9^A^	26.5^A^	29.0^A^	25.7	30.7	27.1^A^	29.2^AB^	26.5^BC^
N_100_PK	32.4^A^	29.7^A^	32.0^A^	29.4	33.3	30.7^A^	33.9^A^	31.1^A^
N_0_PK + Nano-urea	20.7^B^	18.9^B^	20.3^B^	17.8	21.6	19.4^B^	22.1^C^	19.1^D^
N_50_PK+ Nano- urea	26.3^AB^	24.7^AB^	25.8^AB^	23.5	29.6	25.3^AB^	27.0^BC^	24.1^BC^
N_75_PK+ Nano- urea	29.6^A^	28.4^A^	29.1^A^	27.4	31.5	28.8^A^	31.0^AB^	28.2^AB^
N_100_PK+ Nano- urea	30.8^A^	29.7^A^	31.4^A^	29.6	32.5	30.6^A^	33.6^A^	31.0^A^

*Values of means followed by different capital letter(s) (based on Duncan’s multiple range tests) within the row are significantly different at p ≤0.05.

**Table 5:** Effect of nano-urea on dehydrogenase activity (μg TPF g^-1^ 24 hrs^-1^) of maize-wheat and pearl millet-mustard systems.

Treatments	Maize		Wheat		Pearl Millet		Mustard	
	2019–20	2020–21	2019–20	2020–21	2019–20	2020–21	2019–20	2020–21
N_0_PK	20.1^F^	19.2^D^	24.6^C^	24.4^CD^	20.4^C^	23.2^C^	24.4^C^	22.8^C^
N_50_PK	24.3^DE^	22.9^CD^	27.4^BC^	25.2^BCD^	28.3^BC^	28.3^BC^	29.9^BC^	29.8^BC^
N_75_PK	28.9^BC^	27.7^BC^	31.3^ABC^	31.5^ABC^	33.4^AB^	33.4^ABC^	32.9^BC^	30.3^BC^
N_100_PK	33.4^A^	33.8^A^	35.9^AB^	34.4^A^	39.5^A^	39.5^AB^	39.2^AB^	36.9^AB^
N_0_PK + Nano-urea	22.3^EF^	21.4^D^	25.2^C^	23.4^D^	26.5^BC^	26.5^BC^	27.8^C^	27.1^C^
N_50_PK+ Nano- urea	26.7^CD^	24.5^BCD^	30.0^ABC^	29.3^ABCD^	24.8^BC^	25.3^C^	26.3^C^	25.8^C^
N_75_PK+ Nano- urea	31.9^AB^	30.3^AB^	34.8^AB^	32.7^AB^	38.5^A^	38.5^AB^	40.2^AB^	39.7^A^
N_100_PK+ Nano- urea	35.1^A^	34.6^A^	38.3^A^	36.0^A^	42.2^A^	42.2^A^	44.0^A^	43.2^A^

*Values of means followed by different capital letter(s) (based on Duncan’s multiple range tests) within the row are significantly different at p ≤0.05.

**Table 6:** Effect of nano-urea on grain/seed nitrogen uptake (kg ha^-1^) in maize, wheat, pearlmillet and mustard.

Treatment	Maize		Wheat		Pearl millet		Mustard	
	2020	2021	2019–20	2020–21	2020	2021	2019–20	2020–21
N_0_PK	47.9^D^	45.2^D^	38.2^D^	37.3^C^	34.6^D^	32.3^D^	37.8^D^	36.4^E^
N_50_PK	64.8^BC^	63.6^C^	51.7^C^	50.9^B^	47.2^BC^	46.6^BC^	52.3^BC^	50.7^CD^
N_75_PK	73.8^AB^	71.6^BC^	61.0^AB^	58.9^AB^	51.1^AB^	52.8^AB^	61.6^AB^	55.8^BC^
N_100_PK	80.5^A^	81.0^AB^	63.0^AB^	64.5^A^	59.3^A^	62.0^A^	64.7^A^	66.5^AB^
N_0_PK + Nano-urea	52.6^CD^	48.5^D^	42.5^D^	39.9^C^	38.2^CD^	37.6^CD^	47.1^CD^	41.0^DE^
N_50_PK+ Nano- urea	74.2^AB^	74.1^B^	58.3^B^	58.0^AB^	52.8^AB^	52.6^AB^	62.2^AB^	56.4^BC^
N_75_PK+ Nano- urea	76.2^AB^	76.7^AB^	59.3^AB^	57.8^AB^	56.4^AB^	58.3^A^	63.3^A^	61.3^ABC^
N_100_PK+ Nano- urea	87.2^A^	84.7^A^	65.1^A^	65.3^A^	59.7^A^	62.1^A^	65.8^A^	69.7^A^

*Values of means followed by different capital letter(s) (based on Duncan’s multiple range tests) within the row are significantly different at p ≤0.05.

## 3. Results

### 3.1 Productivity

Highest grain yields of maize were recorded under N_100_PK+ nano-urea in the year 2019–20 and 2020–21 (Fig 1). The treatments N_100_PK and N_75_PK+ nano-urea registered comparable grain yields in both cropping seasons. Treatments with applications of 50% of recommended N dose along with nano-urea sprays exhibited significantly lower yields compared with N_100_PK plots. Irrespective of nano-urea applications, all treatments with application of recommended N registered similar grain yields in both cropping seasons. The plots under no fertilizer N application, irrespective of nano-fertilizer spray registered very low grain yields, which were significantly lower compared with recommended NPK application.

Similar yield trends were observed for the wheat crop across seasons. Application of 75% recommended N (90 kg N ha^-1^) + PK along with two sprays of nano-urea recorded significantly higher grain yield (4.93 and 4.91 t ha^-1^ in 2019–20 and 2020–21, respectively) over no nitrogen + PK and remained at par with the recommended dose of fertilizers (100% N *i*.*e*., 120 kg N + PK) (5.02 and 5.14 t ha^-1^ in 2019–20 and 2020–21, respectively) (Fig 1). Application of recommended NPK along with spraying of nano-urea exhibited higher grain yield of wheat. Two sprays of nano-urea along with 50% of recommended N + PK application registered significantly lower grain yields compared with N_100_PK. Whereas, application of nano-urea without N fertilizer application yielded very low grain yields.

Plots treated with 75% recommended N (45 kg N ha^-1^) + PK along with two sprays of nano-urea recorded significantly higher grain yields of pearl millet (3.29 and 3.25 t ha^-1^ in 2019–20 and 2020–21, respectively) compared to recommended NPK application (100% N *i*.*e*., 60 kg N + PK) (Fig 2). Maximum grain yields were obtained under N_100_PK+ nano-urea in both cropping seasons. The treatment N_75_PK+ nano-urea registered ~14% higher grain yields compared with N_50_PK+ nano-urea in both cropping seasons. All the other treatments with 75% recommended N application along with combinations of nano-urea spray also registered significantly higher grain yields compared with 50% recommended N application with nano-fertilizer sprays. Nano-fertilizer spray without recommended N application resulted in significantly lower grain yields, compared with recommended NPK application, with values ranging from 1.37 to 1.44 t ha^-1^.

The mustard seed yields under N_50_PK + nano-urea and N_75_PK + nano-urea were similar in 2019–20 (Fig 2). On the other hand, in 2020–21, treatments with 50% recommended N application with nano-urea sprays registered significantly lower seed yields compared with recommended NPK application. The treatments with 75% recommended N application with nano-urea sprays registered similar seed yields compared with recommended NPK application. In 2020–21, treatment N_75_PK+ nano-urea registered significantly higher seed yield compared with N_50_PK+ nano-urea. Lowest seed yields were found under non-fertilized plots, irrespective of nano-urea applications.

### 3.2 Soil mineral nitrogen

Soil mineral N varied from 16.5 to 31.5 mg kg^-1^ across the treatments and sampling times in the maize-wheat system. Recommended NPK application along with nano-urea spray registered highest values of soil mineral N (31.5 and 30.4 mg kg^-1^ at flowering and post-harvest stages, respectively) in maize crop during 2019–20 (Table 3). During 2020–21, highest mineral N was registered under N_100_PK, and N_100_PK +nano-urea in flowering and post-harvest stages, respectively. In both the years, N_100_PK and N_75_PK+ nano-urea had comparable mineral N in post-harvest soils of maize crop. Overall, the recommended application of NPK had similar soil mineral N across the cropping seasons irrespective of nano-urea application. Treatment N_75_PK+ nano-urea registered ~13% and ~17% lower mineral N compared with N_100_PK in soils at flowering stage of maize crop in 2019–20 and 2020–21, respectively. The N_0_ treatments irrespective of P, K and nano-fertilizer application reported similar mineral N, with values ~41 and 33% lower compared with recommended NPK application and N_75_PK+ nano-urea respectively. In wheat, N_100_PK and N_75_PK+ nano-urea registered similar values of mineral N in flowering and post-harvest soils of 2020–21. In 2019–20, treatments with 50% recommended N doses with sprayings of nano-urea registered significantly lower mineral N compared with recommended NPK application (Table 3). However, the recommended NPK application irrespective of nano-urea application had similar soil mineral N content in wheat crop. Soil mineral N varied from 17.9 to 33.9 mg kg^-1^ across the treatments and sampling times in the pearl millet-mustard system (Table 4). The N_75_PK treatments along with two sprays of nano-urea had similar mineral N as compared with recommended NPK application, irrespective of time of sampling. Treatments without N application, irrespective of nano-urea spray registered ~36% lower mineral N compared with N_75_PK+ nano-urea.

### 3.3 Dehydrogenase activities

Under maize, N_75_PK+ nano-urea registered similar DHA activity (31.9 and 30.3 μg TPF g^-1^ 24 h^-1^ in first and second years, respectively) compared to N_100_PK+ nano-urea (35.1 and 34.6 μg TPF g^-1^ 24h^-1^ in first and second years, respectively) and N_100_PK (33.4 and 33.8 μg TPF g^-1^ 24h^-1^ in first and second years, respectively) (Table 5). On the other hand, N_75_PK+ nano-urea had significantly higher values of DHA, compared with N_50_PK (24.3 and 22.9 μg TPF g^-1^ 24h^-1^ in first and second years, respectively) and N_50_PK+ nano-urea (26.7 and 24.5 μg TPF g^-1^ 24h^-1^ in first and second years, respectively).

Application of recommended N doses (N_100_PK) along with nano-urea spray registered significantly higher DHA (38.3 and 36.0 μg TPF g^-1^ 24h^-1^ in first and second years, respectively) in soil at flowering stage of wheat compared with application of 50% of recommended N doses (Table 5). On the other hand, treatments with application of 75% of recommended N doses registered similar values of DHA as compared with N_100_PK. Under nano-urea spraying treatments, N_100_PK, N_75_PK and N_50_PK had similar DHA, with values significantly higher than that under N_0_PK + nano-urea.

Soil DHA varied from 20.4 to 44.0 μg TPF g^-1^ 24h^-1^ across the treatments and sampling times in the pearl millet-mustard system (Table 5). Irrespective of nano-urea spray, plots received 50% recommended N application registered significantly lower values of DHA compared with those under recommended N application along with nano-urea spray (42.2 and 42.2 μg TPF g^-1^ 24h^-1^ in pearl millet, and 44.0 and 43.2 μg TPF g^-1^ 24h^-1^ in mustard during first and second years, respectively). Application of N_75_PK along with nano-urea spray noted at par DHA (38.5 and 38.5 μg TPF g^-1^ 24h^-1^ in pearl millet, and 40.2 and 39.7 μg TPF g^-1^ 24h^-1^ in mustard during first and second years, respectively) over N_100_PK (39.5 and 39.5 μg TPF g^-1^ 24h^-1^ in pearl millet, and 39.2 and 36.9 μg TPF g^-1^ 24h^-1^ in mustard during first and second years, respectively).

### 3.4 Grain/Seed nitrogen uptake

Grain/seed N uptake was significantly influenced by different treatments of nano-urea under maize-wheat and pearl millet-mustard system (Table 6). The highest grain/seed N uptake was noted under N_100_PK + nano-urea sprays (87.2 and 84.7 kg ha^-1^ for maize, 65.1 and 65.3 kg ha^-1^ for wheat, 59.7 and 62.1 kg ha^-1^ for pearl millet, 65.8 and 69.7 kg ha^-1^ for mustard in first and second years, respectively) and it remained at par with N_75_PK + nano-urea (76.2 and 76.7 kg ha^-1^ for maize, 59.5 and 57.8 kg ha^-1^ for wheat, 56.4 and 58.3 kg ha^-1^ for pearl millet, 63.3 and 61.3 kg ha^-1^ for mustard in first and second years, respectively) and N_100_PK (80.5 and 81.0 kg ha^-1^ for maize, 63.0 and 64.5 kg ha^-1^ for wheat, 59.3 and 62.0 kg ha^-1^ for pearl millet, 64.7 and 66.5 kg ha^-1^ for mustard in first and second years, respectively). Furthermore, N_75_PK + nano-urea was at par with N_100_PK with respect to grain/seed N uptake irrespective of crops.

### 3.5 Profitability

The cost of cultivation with subsidized urea was comparatively lower than unsubsidized urea while net returns and B:C were high across the crops. The cost of cultivation, gross and net returns were highest under N_100_PK + nano-urea in all the tested crops. Significantly (P<0.05) higher net returns (US $ 1044 with and US $ 911 without subsidised urea) and B:C (2.80 with and 2.28 without subsidised urea) were recorded under N_100_PK + nano-urea over N_50_PK (S3 Table in S1 File) while it remained at par with N_75_PK + nano-urea (net return of US $ 911 with and US $ 811 without subsidised urea, and B:C of 2.59 with and 2.21 without subsidised urea) and N_100_PK (net return of US $ 958 with and US $ 825 without subsidised urea, and B:C of 2.75 with and 2.21 without subsidised urea). N_100_PK was again statistically at par with N_75_PK + nano-urea with respect of net returns and B: C.

The N_75_PK with spray of nano-urea recorded similar gross (US $ 1294) and net returns (US $ 787 with and US $ 707 without subsidised urea) with N_100_PK (gross return of US $ 1334, and net return of US $ 852 with and US $ 745 without subsidised urea) for wheat (S4 Table in S1 File). B:C based on subsidised urea was significantly superior under N_100_PK (2.77) than N_75_PK + nano-urea (2.55) but B:C based on unsubsidised urea was statistically at par under N_75_PK + nano-urea treatments (2.21).

The treatment N_100_PK+ nano-urea registered the highest gross (US $ 1309) and net returns (US $ 929 with and US $ 876 without subsidised urea) among the treatments but the same remained at par with N_75_PK + nano-urea (gross return of US $ 1219, and net return of US $ 842 with and US $ 802 without subsidised urea) and N_100_PK (gross return of US $ 1250, and net return of US $ 901 with and US $ 848 without subsidised urea) for pearl millet (S5 Table in S1 File). However, highest B:C (with 3.58 and without 3.11 subsidised urea) was observed under recommended N_100_PK and it was statistically at par with N_75_PK + nano-urea (2.92) when unsubsidized urea was taken into account.

In mustard, highest gross return (US $ 1639) as well as net return (US $ 1240 with and US $ 1169 without subsidised urea) were also obtained under N_100_PK+ nano-urea treatment (S6 Table in S1 File) however, with respect to gross and net returns this treatment was statistically at par with N_75_PK + nano-urea (gross return of US $ 1524, and net return of US $ 1130 with and US $ 1077 without subsidised urea) and N_100_PK treatments (gross return of US $ 1575, and net return of US $ 1207 with and US $ 1136 without subsidised urea). Perusal of the data revealed that by the application of N_100_PK the maximum B:C (4.28 with and 3.59 without subsidised urea) was obtained but, it remained at par with N_75_PK + nano-urea treatment (3.40) while unsubsidized urea was taken into consideration.

### 3.6 Nano-urea additional price/saving

An additional cost ranging from US $ 9.10 to 12.76 was incurred due to application of nano-urea under N_75_PK + nano-urea treatments over N_100_PK across the crops while subsidized urea was taken into account (Table 7). On the other hand, when unsubsidized urea was taken into consideration an additional saving ranging from US $ 7.42 to 41.99 was noted under N_75_PK + nano-urea plot over N_100_PK. Grain/seed yield of maize, wheat, pearl millet and mustard under N_75_PK + nano-urea treatment was at par with N_100_PK.

**Table 7:** Effect of nano-urea on additional price and saving in different crops.

Treatments	N Dose (kg ha^-1^)	Urea Equivalent (kg ha^-1^)	Nano-urea Cost [(US $ 3.03/500 ml), [Total requirement for 1 ha 2500 ml]	Subsidized Urea Cost [@ US $ 3.36/Bag (one bag = 45 kg urea)	Total Cost in US $ (Urea + nano-urea)	Additional cost in Nano Plot (US $)	Total cost of Non-Subsidized Urea (@ US $ 28.38 per 45 kg bag)	Total Cost in US $ (unsubsidized urea cost + nano-urea)	Additional saving due to nano-urea application (US $/ha) on non-subsidized urea	#Grain Yield (t ha^-1^)
**Maize**										
N_100_PK	150	326	-	24.3	24.3	-	205.6	205.6	-	6.00
N_75_PK+ Nano-urea	112.5	245	15.2	18.3	33.4	9.10	154.5	163.6	42.0	5.92
**Wheat**										
N_100_PK	120	261	-	19.5	19.5	-	164.6	164.6	-	5.08
N_75_PK+ Nano-urea	90	196	15.2	14.6	29.8	10.3	123.6	133.9	30.7	4.92
**Pearl millet**										
N_100_PK	60	130	-	9.7	9.7	-	82.0	82.0	-	3.34
N_75_PK+ Nano-urea	45	98	15.2	7.3	22.5	12.8	61.8	74.6	7.42	3.27
**Mustard**										
N_100_PK	80	174	-	13.0	13.0	-	109.7	109.7	-	2.30
N_75_PK+ Nano-urea	60	130	15.2	9.71	24.9	11.9	82.0	93.9	15.9	2.22

^#^Yield was statistically at par

### 3.7 Energy efficiency

Based on the energy equivalents (S2 Table in S1 File), energy input, energy output, net energy returns and energy use efficiency (Table 8) were calculated. Energy input was highest under N_100_PK + nano-urea across the different crops under the investigation. Energy outputs under N_100_PK + nano-urea treatments (241997, 167846 and 162131 MJ ha^-1^ under maize, wheat and pearl millet, respectively) were significantly superior over rest of the treatments except in pearl millet where it remained at par with N_100_PK.

**Table 8:** Effect of nano-urea on energetics under different crops (based on mean of two years).

Treatments	Energy input (MJ ha^-1^)				Energy output (MJ ha^-1^)				Net Energy Returns (MJ ha^-1^)				Energy Use efficiency (%)			
	Maize	Wheat	Pearl millet	Mustard	Maize	Wheat	Pearl millet	Mustard	Maize	Wheat	Pearl millet	Mustard	Maize	Wheat	Pearl millet	Mustard
N_0_PK	11975	13074	9638	10508	131918^F^	88311^F^	95383^F^	98164^F^	119943^F^	75237^F^	85745^F^	87656^G^	11.0^CD^	6.8^D^	9.9^C^	9.3^F^
N_50_PK	16520	16710	11456	12932	177655^D^	123147^D^	126008^D^	131073^D^	161135^D^	106437^D^	114552^D^	118141^E^	10.7^D^	7.4^C^	11.0^B^	10.1^E^
N_75_PK	18792	18528	12365	14144	205153^C^	146413^C^	134492^C^	148870^C^	186361^C^	127885^C^	122127^C^	134726^D^	10.9^D^	7.9^ABC^	10.9^B^	10.5^DE^
N_100_PK	21065	20346	13274	15356	228741^B^	159074^AB^	155635^AB^	163265^AB^	207676^B^	138728^AB^	142361^AB^	147909^AB^	10.8^D^	7.8^BC^	11.7^A^	10.6^CDE^
N_0_PK + Nano-urea	11985	13085	9649	10519	148821^E^	102725^E^	106498^E^	113683^E^	136836^E^	89640^E^	96849^E^	103164^F^	12.4^A^	7.8^BC^	11.0^B^	10.8^BCD^
N_50_PK+ Nano- urea	16530	16721	11467	12943	201134^C^	138841^C^	136323^C^	151074^C^	184604^C^	122120^C^	124856^C^	138131^CD^	12.2^AB^	8.3^AB^	11.9^A^	11.7^A^
N_75_PK+ Nano- urea	18803	18539	12376	14155	221860^B^	155833^B^	151097^B^	160364^B^	203057^B^	137294^B^	138721^B^	146209^BC^	11.8^ABC^	8.4^A^	12.2^A^	11.4^AB^
N_100_PK+ Nano- urea	21075	20357	13285	15367	241997^A^	167846^A^	162131^A^	171470^A^	220922^A^	147489^A^	148846^A^	156103^A^	11.5^BCD^	8.3^AB^	12.2^A^	11.1^ABC^

*Values of means followed by different capital letter(s) (based on Duncan’s multiple range tests) within the row are significantly different at p ≤0.05.

Significantly higher net energy returns (220922, 147489, 148846 and 156103 MJ ha^-1^ under maize, wheat, pearl millet and mustard, respectively) were recorded under N_100_PK + nano-urea over rest of the treatments and remained at par with N_100_PK in wheat (138728 MJ ha^-1^), pearl millet (142361 MJ ha^-1^) and mustard (147909 MJ ha^-1^). The treatment N_75_PK + nano-urea recorded significantly higher energy use efficiency (11.8, 8.4, and 11.4 under maize, wheat and mustard, respectively) over N_100_PK (Table 8). While, energy use efficiency under N_75_PK + nano-urea treatment (12.2) was statistically at par with N_100_PK (11.7) and N_100_PK + nano-urea (12.2).

### 3.8 Greenhouse gas emission (GHG)

Greenhouse gas emission influenced by 2 treatments (N_100_PK and N_75_PK + nano-urea) was quantified and presented in Table 9. The GHG emission under N_100_PK was high (1678.9, 1344.2, 669.5 and 896.1 kg CO_2_-eq ha^-1^ in maize, wheat, pearl millet and mustard, respectively) while 25% reduction in N doses along with nano-urea spray (N_75_PK + nano-urea) recorded less GHG emission (1262.4, 1010.0, 505.3 and 670.1 kg CO_2_-eq ha^-1^ in maize, wheat, pearl millet and mustard, respectively). The reduction in GHG emission due to nano-urea application (N_75_PK + nano-urea) was 416.5, 334.1, 164.2 and 226.0 kg CO_2_-eq ha^-1^ in maize, wheat, pearl millet and mustard, respectively.

**Table 9:** Effect of nano-urea on greenhouse gas emission (GHG).

Treatments	Nitrogen Dose (kg ha^-1^)	Urea Equivalent (kg ha^-1^)	Nano-urea application per ha (ml)	GHG emission (kg CO_2_-eq perha from urea)	GHG emission(kg CO_2_-eq per ha from nano-urea)	Total GHG emission (kg CO_2_-eq per ha)	Reduction in GHG emission (kg CO_2_-eq per ha) due to nano-urea application
**Maize**							
N_100_PK	150	326	-	1678.9	-	1678.9	-
N_75_PK+ Nano-urea	112.5	245	2500	1261.8	0.62	1262.4	416.5
**Wheat**							
N_100_PK	120	261	-	1344.2	-	1344.2	-
N_75_PK+ Nano-urea	90	196	2500	1009.40	0.62	1010.0	334.1
**Pearl millet**							
N_100_PK	60	130	-	669.5	-	669.5	-
N_75_PK+ Nano-urea	45	98	2500	504.7	0.62	505.3	164.2
**Mustard**							
N_100_PK	80	174	-	896.1	-	896.1	-
N_75_PK+ Nano-urea	60	130	2500	669.5	0.62	670.1	226.0

## 4. Discussion

### 4.1 Productivity and N uptake

Application of 75% recommended dose of N + PK along with two sprays of nano-urea recorded statistically at par results with N_100_PK for the yields of maize, wheat, pearl millet and mustard during both the years (Figs 1 and 2). Although the yields of wheat and mustard were statistically at par during first year, yield of both crops significantly decreased under N_75_PK than N_100_PK during second study year. This might be due to decline of inherent fertility status of the soil which supplemented the N nutrition to both the crops during first year (the year of start of the experiment). Whereas, the application of nano-urea had advantage over no application of nano-urea in the same treatment (N_75_PK). Hence, up to 25% of recommended N dose can be curtailed without any yield penalty, with nano-urea application. In the current study, nano-urea was sprayed on leaves, leading to direct penetration through stomatal pores, and transportation through plasmodesmata [25]. Diminutive surface property and size of nano-urea enable its penetration into the plants through leaves. After entry in plant systems, nano-urea releases N in a controlled manner. The uptake efficacy of nano-urea by plant is 80% higher [25] than conventional urea. However, the efficiency of nano-urea depends on the concentration, method of application and weather conditions. Babu et al. [17] opined that, efficiency of nano-urea was higher under warm weather condition due to better acquisition and translocation of nano-urea by plants.

Nano-urea boosts speedy nutrients availability to growing plant parts, ensuing increased dry matter accumulation, chlorophyll production, plant growth, development (data not reported) and yield. Nitrogen is an important structure of chlorophyll and higher N content can positively influence chlorophyll biosynthesis [42] which in turn can reflect as increased photosynthesis and subsequent increase in dry matter accumulation, this can explain the increased yield seen in the foliar applied Nano N treatment. The enhancement in the crop yield is possibly due to the synchronous release of N from the nano-urea following the demand of the crops (maize, wheat, pearl millet and mustard). Smart release of N from nano-urea improved photosynthesis by acquiring sufficient light-harvesting chlorophyll-protein complexes and also did not cause any stress in the crops resulting in the enhanced growth and ultimately yield [17]. Nano-urea discharges nutrients in 40–50 days [24], and it is applied on the leaves instead of soil; whereas conventional urea is applied in soil and discharges nutrients in 2–7 days [43]. Leaching and volatilization accounts more than 70% of applied conventional urea and leaving only <20% [44] readily available for plants growth. Nano-urea releases nitrogen 12 times slower than urea and thus is available for functional metabolic interaction for a longer time and this can be one of the reasons for increased grain yields [21].

Our results are consistent with Kumar et al. [24] who reported that foliar sprays of nano-fertilizer at critical crop growth stages either in isolation or in combination with fertilizers increases crop yields even at reduced levels of application of their conventional analogues. Al-Juthery et al. [27] and Abdel-Aziz et al. [45] indicated that foliar spray of nano-fertilizers significantly improved the plant growth characteristics and yield of wheat. Yield attributes viz., number of effective tillers per metre row length, ear length (cm), grains per ear, test weight etc. of crop were also higher in nano-fertilizer applied plots [46]. Nano-NPK applications were reported to stimulate the porphyrin molecules present in metabolic compounds, in turn, increasing plant biomass, yield and yield attributes of maize [47, 48]. Irrespective of crops, N uptake under N75PK + nano-urea treatment was at par with N100PK (Table 6). This was mainly due to the statistically similar level of yield observed in all crops under said treatments vis-à-vis statistically at par N content was recorded under both the treatments. It indicates that uptake mechanism is also triggered by the application of nano-urea as foliar spray. Similar finding was also reported by [17, 48].

Nano-urea contains nanoscale particles of 18–30 nm size range, which has ~ 10,000 times more surface area compared with conventional prilled urea [24]. Thus, the nano-urea particles have high surface area to volume ratio, unique magnetic properties, electronic states and catalytic reactions [19]. These unique properties manifest in exceptionally high reactivity of nano-fertilizers compared with conventional fertilizers [19, 48]. The nano particles have the ability of binding with carrier proteins through aquaporin and ion channels, and in turn, tend to facilitate different metabolic processes in plant systems, resulting in higher production of photosynthates [24]. Furthermore, nano-fertilizers mediate nutrient absorption in synchrony with plant demand for nutrients [49]. The yield enhancement due to Nano-fertilizers were reported in wheat [26–28] maize [29] and Indian mustard [30–32].

### 4.2 Soil mineral nitrogen

Results indicated the possibility of curtailing up to 25% of recommended N dose through nano-urea spray (Tables 3 and 4). The nano-urea contains ~ 4% N by weight [24], which by itself cannot possibly supply the remaining ~ 25% of recommended N dose to plants. As the experimental soil is deficient in N, we obtained yield responses to nano-urea as well as N fertilizer application in both cropping systems. Nano-urea application possibly enhanced root morphology and growth [50]. As a result, use efficiency of native soil N and applied N increased by virtue of greater nutrient adsorption surface of the crops [50]. Application of nano-urea tapped into the vast pool of non-mineral (apart from ammonium and nitrate) N present in soil which is ~96–98% of total soil N. Therefore, the treatments N_100_PK and N_75_PK+nano-urea registered similar values of mineral N across the seasons (Tables 3 and 4), implying non-occurrence of nutrient mining. Nonetheless, the recommended N applications along with nano-urea spray reported highest soil mineral N. Further, 50% of recommended N applications depleted soil mineral N, irrespective of nano-urea applications. On the other hand, treatments with no N applications recorded very less mineral N across the seasons even with the nano-urea spray. Therefore, at least 75% of the recommended N should be applied to avoid N mining.

### 4.3 Dehydrogenase activities (DHA)

The N application is directly linked with greater shoot and root biomass production [51]. Enhanced root growths under recommended N applied plots might have caused greater production of root exudates and other secretions. These carbonaceous materials often act as substrates for microbial growth and metabolism. Therefore, the recommended N applied plots registered highest values of DHA, which supports this contention (Table 5) [52]. Further, DHA is considered to be proportional to the biomass of the microorganisms present in the soil [52]. The application of nano-urea further stimulated root growth and activities, in turn favouring soil enzymatic activities. On the other hand, 50% curtailing of N fertilizer doses, irrespective of nano-spray registered significant decline in crop yield (Figs 1 and 2) *vis-à-vis* root growth and activities. The decline in root growth in turn registered significant decline in DHA in N_50_ and N_0_ plots. The nano-urea application did not register any negative impact on soil microbial health.

### 4.4 Profitability and additional price/saving

The cost of cultivation with unsubsidized urea was comparatively higher than subsidized urea while net returns and B:C were lower across the crops (S3-S6 Tables in S1 File). This is due to high cost of unsubsidized urea (US $ 28.38 per 45 bag) where cost of subsidized urea was US $ 3.36. As far as urea-price is concerned government of India (GoI) is giving huge subsidy on it. According to Fertilizer Statistics 2019–20 [53] total requirement of N source as urea was 33.9 mt (24.8 mt produced in India and 9.1 mt imported from different countries) and government provided US $ 478 billion subsidies to the Indian Farmers. That indicates whether we should enhance the efficiency of urea or should have some alternatives as novel fertilizers to cut the additional economic burden on government as well as on farmers.

Overall, the economics in terms of net returns and B:C under N_75_PK + nano-urea treatment over N_100_PK were almost similar across the different crops during both the years (S3-S6 Tables in S1 File). This indicates application of two sprays of nano-urea can curtail up to 25% of the recommended dose of N. In this way out of total requirement of urea (33.9 mt) in the country like India 8.5 mt of its use can be replaced with nano-urea which can save all the subsidised cost borne by importing urea of around US $ 384 billion. Although, the spraying cost of nano-urea incurred additional burden on farmers but it can be minimized using drone on custom hiring basis.

Application of nano-urea along with N_75_PK (Table 7) based on subsidised urea incurred additional cost ranging from US $ 9.10 to 12.76 per ha over recommended dose of nitrogen (N_100_PK) under different test crops used for the study. This is due to huge subsidy on urea fertilizer provided by the government of India. In contrast, N_75_PK along with 2 times nano-urea sprays resulted additional saving ranges from US $ 7.42 to 41.99 based on unsubsidized urea price. The saving was more in case of maize where higher recommended dose of N (150 kg ha^-1^) was applied while less saving was noted in pearl millet where recommended N dose was less i.e., 60 kg ha^-1^. The maximum B:C ratio under 50% recommended NPK application along with nano-urea spray were reported by [24, 54].

### 4.5 Energy efficiency

Energy input was highest under N_100_PK + nano-urea across the four crops under the investigation followed by N_100_PK and the difference between these two treatments was only 11 MJ ha^-1^ due to addition of nano-urea. It indicates energy requirement for the production of nano-urea is very less or in other words it is a relatively energy efficient technology. As energy equivalent for production of one bottle of nano-urea is 2.13 MJ ha^-1^ per 500 mL. In this way total 5 bottles of nano-urea (needed for 2 sprays in 1 ha) required 10.65 MJ ha^-1^ energy. One the other hand, energy equivalent for the production of 1 kg N is 60.6 MJ ha^-1^. In this way energy input required for supplementing N in maize, wheat, pearl millet and mustered is 9090, 7272, 3636 and 4848 MJ ha^-1^. Therefore, curtailing 25% demand of N in crops by the intervention of nano-urea would be one of the best energy saving practices. Energy use efficiency is one of the indicators which show the potential of any production system in terms of its effectiveness [17]. Based on the results of this parameter N_75_PK + nano-urea was found to be an energy efficient option over N_100_PK irrespective of crops. This is due to statistically at par yields among all crops were noticed under N_75_PK + nano-urea over N_100_PK along with reduction of 25% dose of N as observed in the former case over latter.

### 4.6 Greenhouse gas emission (GHG)

Greenhouse gas emission due to treatments (N_100_PK and N_75_PK+ nano-urea) was calculated using reference values of urea production + use (5.15 kg CO_2_-eq kg^-1^ product) [6]. GHG emission reference value 0.248 kg CO_2_-eq L^-1^ product was taken into consideration for nano-urea. Comparatively higher GHG emission was recorded in N_100_PK treatments over N_75_PK + nano-urea due to 25% reduction in nitrogen doses because per kg production of urea emits more GHG over nano-urea.

## 5. Conclusion

Based on the present investigation, it can be inferred that conjoint application of nano-urea with conventional nitrogen fertilizer have potential to enhance crop yields, nutrient uptake, and lowering GHG emissions. Application of 75% recommended N through conventional N fertilizer (urea) in maize (three equal split), wheat (three equal split), pearl millet (two equal split) and mustard (two equal split) + full dose of P_2_O_5_ and K_2_O along with 2 time sprays of nano-urea (1250 ml ha^-1^ spray^-1^) resulted statistically at par grain yield over recommended dose of fertilizer (100%N + full dose of P_2_O_5_ and K_2_O). Therefore, there is a possibility of curtailing up to 25% of the recommended dose of N by application of two sprays of nano-urea in maize-wheat and pearl millet-mustard cropping systems was observed. In this way, nano-urea seemed to have the potential to bring forth paradigm shift in the fertilizer consumption scenario of India and world. However, the findings of the nano-urea need to be further validated across the crops and locations before reaching to the policy.

## Supporting information

S1 File (DOCX)

S1 Graphical abstract (TIF)

## References

[1] United Nations Department of Economic and Social Affairs, Population Division, Global Population Growth and Sustainable Development. 2021. UN DESA/POP/2021/TR/NO.2.

[2] FAO. The state of the world’s land and water resources for food and agriculture–Systems at breaking point. Synthesis report. 2021. Rome.

[3] Abebe TG, Tamtam MR, Abebe AA, Abtemariam KA, Shigut TG, Dejen YA, et al. Growing Use and Impacts of Chemical Fertilizers and Assessing Alternative Organic Fertilizer Sources in Ethiopia. Appl. Environ. Soil Sci. 2022; 14. doi: 10.1155/2022/4738416

[4] Wen P, Wu Z, Han Y, Cravotto G, Wang J, Ye BC. Microwave-assisted synthesis of a novel biochar-based slow-release nitrogen fertilizer with enhanced water-retention capacity. ACS Sustain. Chem. Eng. 2017; 5:7374–7382. doi: 10.1021/acssuschemeng.7b01721

[5] FAO, World fertilizer trends and outlook to 2022, Rome, 2019.

[6] Parry ML, Canziani OF, Palutikof JP, Linden van der PJ, Hanson CE, (Eds.), Impacts, Adaptation and Vulnerability, Climate Change, Contribution of Working Group II to the Fourth Assessment Report of the Intergovernmental Panel on Climate Change, IPCC, Cambridge University Press, Cambridge, 2007, pp. 976.

[7] Fiamelda L, Suprihatin Purwoko. Analysis of water and electricity consumption of urea fertilizer industry: case study PT. X. Earth Environ. Sci. 2020; 472:012034. doi: 10.1088/1755-1315/472/1/012034

[8] Bartolucci B, Scognamiglio V, Antonacci A, Fraceto LF. What makes nanotechnologies applied to agriculture green?. Nano Today. 2022; 43:1–5. doi: 10.1016/j.nantod.2022.101389

[9] Al-Juthery HW, Lahmod NR, Al-Taee RA. Intelligent, Nano-fertilizers: A New Technology for Improvement Nutrient Use Efficiency (Article Review). Earth. Environ. Sci. 2021; 735:1–012086. doi: 10.1088/1755-1315/735/1/012086

[10] Pachauri RK, Meyer (Eds.) LA. Contribution of Working Groups I, II and III to the Fifth Assessment Report of the Intergovernmental Panel on Climate Change, Core Writing Team, IPCC, Geneva, Switzerland. 2014; 151.

[11] Mahmud K, Panday D, Mergoum A, Missaoui A. Nitrogen Losses and Potential Mitigation Strategies for a Sustainable Agroecosystem. Sustain. 2021; 13:2400. doi: 10.3390/su13042400

[12] Guardia G, Sanz-Cobena A, Sanchez-Martín L, Fuertes-Mendizábal T, González-MuruabJosé C, Álvarez JM, et al. Urea-based fertilization strategies to reduce yield-scaled N oxides and enhance bread-making quality in a rainfed Mediterranean wheat crop. Agric. Ecosyst. Environ. 2018; 265:421–431. doi: 10.1016/j.agee.2018.06.033

[13] Verma KK, Song XP, Joshi A, Tian DD, Rajput VD, Singh M, et al. Recent Trends in Nano-Fertilizers for Sustainable Agriculture under Climate Change for Global Food Security. Nanomaterials. 2022; 12:173. doi: 10.3390/nano12010173 35010126 PMC8746782

[14] Kah M, Kookana RS, Gogos A, Bucheli TD. A critical evaluation of nanopesticides and nanofertilizers against their conventional analogues. Nat. Nanotechnol. 2018; doi: 10.1038/s41565-018-0131-1 29736032

[15] Hua J, Xianyu Y. When nano meets plants: A review on the interplay between nanoparticles and plants. Nano Today 2021; 3:1–20. doi: 10.1016/j.nantod.2021.101143

[16] Angle JS. Expectations from nano in agriculture. Nat. Nanotechnol. 2019; 14:515–516. doi: 10.1038/s41565-019-0471-5 31168082

[17] Babu S, Singh R, Yadav D, Rathor SS, Raj R, Avasthe R, et al. Nanofertilizers for agricultural and environmental sustainability. Chemosphere. 2022; 292:1–19. doi: 10.1016/j.chemosphere.2021.133451 34973251

[18] Kottegoda N, Sandaruwan C, Priyadarshana G, Siriwardhana A, Rathnayake UA, Arachchige DMB, et al. Urea-Hydroxyapatite Nanohybrids for Slow Release of Nitrogen. ACS Nano. 2017; 11:1214–1221. doi: 10.1021/acsnano.6b07781 28121129

[19] Raliya R, Saharan V, Dimpka C, Biswas P. Nanofertilizer for Precision and Sustainable Agriculture: Current State and Future Perspectives. Agric J. Food Chem. 2018; 66:6487–6503. doi: 10.1021/acs.jafc.7b02178 28835103

[20] Iqbal MA. Nano-Fertilizers for Sustainable Crop Production under Changing Climate: A Global Perspective. Sustain. Crop Prod. 2020; doi: 10.5772/intechopen.89089

[21] Saurabh K, Manjaiah KM, Datta SC, Thekkumpurath AS, Kumar R. Nanoclay polymer composites loaded with urea and nitrification inhibitors for controlling nitrification in soil. Arch. Agron. Soil Sci. 2019; 65:478–491. doi: 10.1080/03650340.2018.1507023

[22] Rahale S. Nutrient release pattern of nanofertilizer formulation, PhD (Agri.) Thesis, Tamilnadu Agricultural University, Coimbatore. 2011.

[23] Qayyum MF, Abdullah MA, Rizwan M, Haider G, Ali MA, Zafar-ul-Hye M, et al. Different nitrogen and biochar sources’ application in an alkaline calcareous soil improved the maize yield and soil nitrogen retention. Arab. J. Geosci. 2019; 12:1–10. doi: 10.1007/s12517-019-4846-6

[24] Kumar Y, Singh T, Raliya R, Tiwari KN. Nano Fertilizers for Sustainable Crop Production, Higher Nutrient Use Efficiency and Enhanced Profitability. Indian J. Fert. 2021a; 17:1206–1214.

[25] Kumar Y, Tiwari KN, Singh T, Raliya R. Nanofertilizers and their role in sustainable agriculture. Ann. plant soil res. 2021b; 23:238–255. doi: 10.47815/apsr.2021.10067

[26] Behboudi F, Tahmasebi Sarvestani Z, Zaman Kassaee M, Modares Sanavi SAM. Improving growth and yield of wheat under drought stress via application of SiO_2_ nanoparticles. J. Agric. Sci. Technol. 2018; 20:1479–149.

[27] Al-Juthery HWA, Hardan H. M, Al-Swedi FG, Obaid MH, Al-Shami QMN. Effect of foliar nutrition of nano-fertilizers and amino acids on growth and yield of wheat. Earth Environ. Sci. 2019; 388:012046. doi: 10.1088/1755-1315/388/1/012046

[28] Du W, Yang J, Peng Q, Liang X, Mao H. Comparison study of zinc nanoparticles and zinc sulphate on wheat growth: from toxicity and zinc biofortification Chemosphere. 2019; 227109–116. doi: 10.1016/j.chemosphere.2019.03.168 30986592

[29] Manikandan A, Subramaniam KS. Evaluation of zeolite-based nitrogen Nano-fertilizers on maize growth, yield and quality on inceptisol and alfisols. Int. J. Plant Soil Sci. 2018; 9:1–9, doi: 10.9734/IJPSS/2016/22103

[30] Arora S, Sharma P, Kumar S, Nayan R, Khanna PK, Zaidi MGH. Gold-nanoparticle induced enhancement in growth and seed yield of Brassica juncea. Plant Growth Regul. 2012; 66:303–310. doi: 10.1007/s10725-011-9649-z

[31] Das A, Babu S, Yadav GS, Ansari MA, Singh R, Baishya LK, et al. Status and strategies for pulses production for food and nutritional security in North-Eastern Region of India. Indian J. Agron. 2016; 61:43–47.

[32] Rathore SS, Singh RK, Shekhawat K, Upadhyay PK, Shekhawat R, Premi OP. Effect of nano-particles on growth, productivity, profitability of Indian mustard (Brassica juncea) under semi-arid conditions. Indian J. Agric. Sci. 2019; 89:1145–50.

[33] Casida LE. Microbial metabolic activity in soil as measured by dehydrogenase determinations. Appl Jr. Environ. Microbiol. 1977; 34:630–636. doi: 10.1128/aem.34.6.630-636.1977 339829 PMC242722

[34] Rowell DL. Soil science: Methods & Applications Addison Wesley Longman Singapore Publishers (Pte) Ltd. England, UK, 1994; 350.

[35] Kjeldahl J. Neue Methode zur Bestimmung des Stickstoffs in organischen Korpern. Zeitschrift für Analytische Chemie 1883; 22:366–382, 10.1007/BF01338151

[36] Lindsay WL, Norvell WA. Development of a DTPA Soil Test for Zinc, Iron, Manganese, and Copper. Soil Sci. Soc. Am. J. 1978; 42:421–428. doi: 10.2136/sssaj1978.03615995004200030009x

[37] Jackson ML. Methods of Chemical Analysis Prentice Hall of India (Pvt.) Ltd., New Delhi 1973.

[38] Black CA. Soil-Plant Relationship Wiley & Sons Publication, New York, USA, 1967; 515–516.

[39] Upadhyay PK, Sen A, Singh Y, Singh RK, Prasad SK, Sankar A, et al. Soil Health, Energy Budget, and Rice Productivity as Influenced by Cow Products Application with Fertilizers Under South Asian Eastern Indo-Gangetic Plains Zone. Front. Agron. 2022; 37:58572. doi: 10.3389/fagro.2021.758572

[40] Devasenapathy P, Senthilkumar G, Shanmugam PM. Energy management in crop production. Indian J. Agron. 2009; 54:80–90.

[41] Gupta SC, Kapoor VK. Fundamentals of applied statistics Sons publishing house. 2012; 6:94–695.

[42] Sun J, Li W, Li C, Chang W, Zhang S, Zeng Y, et al. Effect of Different Rates of Nitrogen Fertilization on Crop Yield, Soil Properties and Leaf Physiological Attributes in Banana Under Subtropical Regions of China. Front. Plant Sci. 2020; 11:6137601. doi: 10.3389/fpls.2020.613760 33408734 PMC7779679

[43] Seleiman MF, Al Suhaibani N, Ali N, Akmal M, Alotaibi M, Refay Y, et al. Drought Stress Impacts on Plants and Different Approaches to Alleviate Its Adverse Effects. Plants. 2021; 10:1–25. doi: 10.3390/plants10020259 33525688 PMC7911879

[44] Kahrl F, Li Y, Su Y, Tennigkeit T, Wilkes A, Xu J. Greenhouse gas emissions from nitrogen fertilizer use in China. Environ. Sci. Policy. 2010; 138:688–694.

[45] Abdel-Aziz HMM, Hassaneen MNA, Omer AM. Nano chitosan-NPK fertilizer enhances the growth and productivity of wheat plants grown in sandy soil. Span. J. Agric. Res. 2016; 14:1–9. doi: 10.5424/sjar/2016141-8205

[46] Wu M. Effects of Incorporation of Nano-carbon into Slow-released Fertilizer on Rice Yield and Nitrogen Loss in Surface Water of Paddy Soil. Adv. J. Food Sci. Technol. 2013; 5:398–403. 10.19026/ajfst.5.3278

[47] Alzreejawi SAM, Al-Juthery HWA. Effect of Spray with Nano NPK, Complete Micro Fertilizers and Nano Amino Acids on Some Growth and Yield Indicators of Maize (Zea mays L.). Earth. Environ. Sci. 2019; 553:012010, doi: 10.1088/1755-1315/553/1/012010

[48] Grillo R, Mattos BD, Antunes DR, Forini MML, Monikh FA, Rojas OJ. Foliage adhesion and interactions with particulate delivery systems for plant nanobionics and intelligent agriculture. Nano Today. 2021; 37:1–20. doi: 10.1016/j.nantod.2021.101078

[49] Tarafdar JC, Sharma S, Raliya R. Nanotechnology: Interdisciplinary science of applications. Afr. J. Biotechnol. 2012; 12:219–226. doi: 10.5897/AJB12.2481I

[50] Tarafdar JC, Raliya R, Mahawar H, Rathore I. Development of Zinc Nanofertilizer to Enhance Crop Production in Pearl Millet (Pennisetum americanum). Agribiol. Res. 2014; 3:257–262. doi: 10.1007/s40003-014-0113-y

[51] Qi D, Hu T, Song X, Zhang M. Effect of nitrogen supply method on root growth and grain yield of maize under alternate partial root zone irrigation. Sci. Rep. 2019; 9:8191. doi: 10.1038/s41598-019-44759-2 31160666 PMC6546698

[52] Ghosh A, Bhattacharyya R, Dey A, Dwivedi BS, Meena MC, Manna MC, et al. Long-term fertilisation impact on temperature sensitivity of aggregate associated soil organic carbon in a sub-tropical inceptisol. Soil Till. Res. 2019; 195:104369. doi: 10.1016/j.still.2019.104369

[53] Fertilizer Statistics, Fertilizer Association of India, 67^th^ ed., New delhi, India, 2021.

[54] Ajithkumar K, Kumar Y, Savitha AS, Ajayakumar MY, Narayanaswamy C, Raliya R, et al. Effect of IFFCO Nanofertilizer on Growth, Grain Yield and Managing Turcicum Leaf Blight Disease in Maize. Int. J. Plant Soil Sci. 2021; 33:19–28. doi: 10.9734/ijpss/2021/v33i1630519

