# Supplementary material for: Conjoint application of nano-urea with conventional fertilizers: An energy efficient and environmentally robust approach for sustainable crop production
Source: PLoS One. 2023 Jul 5;18(7):e0284009. doi: 10.1371/journal.pone.0284009 (PMC10321634; doi:10.1371/journal.pone.0284009)
Supplement: S1 File — (DOCX) [file pone.0284009.s001.docx]

**Supporting information**

**Integration of nano-urea with conventional fertilizers: An energy efficient and environmentally robust approach for sustainable crop production**

Pravin Kumar Upadhyay^1^, Abir Dey^2^, Vinod Kumar Singh^1,3*^, Brahma Swaroop Dwivedi^2,4^, Tarunendu Singh^5^, Rajanna, G.A^1,6^., Subhash Babu^1^, Sanjay Singh Rathore^1^, Rajiv Kumar Singh^1^, Kapila Shekhawat^1^, Meenakshi^1,7^, Pradeep Kumar^8^, Dhinu Yadav^2^, Devendra Pratap Singh^9^, Debarshi Dasgupta^2,10^, and Gaurav Shukla^1^

^1^Division of Agronomy, ICAR-Indian Agricultural Research Institute, New Delhi-110 012, India

^2^Division of Soil Science and Agricultural Chemistry, ICAR-Indian Agricultural Research Institute, New Delhi-110 012, India

^3^ICAR-Central Research Institute for Dryland Agriculture, Hyderabad- 500 059, India

^4^Agricultural Scientists Recruitment Board, 110 012, India

^5^Indian Farmers Fertiliser Cooperative Limited, New Delhi-110 017, India

^6^ICAR- Directorate of Groundnut Research, Regional Station- Ananthapur-515 701, India

^7^GD Goenka University, Haryana-122 103, India

^8^Nano Fertilizer Plant, Indian Farmers Fertiliser Cooperative Limited, Prayagraj-212404, India

^9^Chandra Shekhar Azad University of Agriculture & Technology, Kanpur-682 022, India

^10^North Dakota State University, Fargo, ND-58105, USA

***Corresponding author:**

Vinod Kumar Singh [vkumarsingh_01@yahoo.com]; Abir Dey [abirdey21@gmail.com]; Rajanna G.A. [rajanna.ga6@gmail.com]; Sanjay Singh Rathore [sanjayrathorears@gmail.com]

**Contents of this file**

Supplementary Table: Table S1-S6

Table S1. Time of application of nano-urea in different crops

| S.No. | Crops | Date of sowing | *1^st^ spray* | *2^nd^ spray* | *Flowering* |
| --- | --- | --- | --- | --- | --- |
| 1 | Wheat (1^st^ year) | 08-11-2019 | 07-12-2019 | 07-01-2020 | 17-01-2020 |
| 2 | Wheat (2^nd^ year) | 05-11-2020 | 06-12-2020 | 11-01-2021 | 18-01-2021 |
| 3 | Mustard (1^st^ year) | 30-10-2019 | 06-12-2019 | 24-12-2019 | 30-12-2019 |
| 4 | Mustard (2^nd^ year) | 27-10-2020 | 04-12-2020 | 24-01-2021 | 30-12-2021 |
| 5 | Maize (1^st^ year) | 11-07-2020 | 11-08-2020 | 04-09-2020 | 10-09-2020 |
| 6 | Maize (2^nd^ year) | 16-07-2021 | 16-08-2021 | 09-09-2021 | 13-09-2020 |
| 7 | Pearl millet (1^st^ year) | 23-07-2020 | 23-08-2020 | 13-09-2020 | 17-09-2020 |
| 8 | Pearl millet (2^nd^ year) | 16-07-2021 | 18-08-2021 | 09-09-2021 | 13-09-2021 |

Table S2. Energy equivalent of inputs and outputs

| Particulars | Units | Equivalent energy (MJ) | Reference |
| --- | --- | --- | --- |
| Human Labour (Adult men) | hour | 1.96 | Devasenapathy et al, 2009 |
| Human Labour (Women) | hour | 1.57 | Devasenapathy et al, 2009 |
| Diesel | litre | 56.31 | Devasenapathy et al, 2009 |
| Farm machinery | kwh | 11.93 | Devasenapathy et al, 2009 |
| Chemical fertilizers |  |  |  |
| N | kg | 60.6 | Devasenapathy et al, 2009 |
| P_2_O_5_ | kg | 11.1 | Devasenapathy et al, 2009 |
| K_2_O | kg | 6.70 | Devasenapathy et al, 2009 |
| Water for irrigation | m^3^ | 1.02 | Devasenapathy et al, 2009 |
| Plant protection (Superior) | kg | 120 | Devasenapathy et al, 2009 |
| Grain/seed | kg | 14.7 | Devasenapathy et al, 2009 |
| Straw/stover  Nano-urea | Kg  Per bottle  (500 ml) | 12.5  2.13 | Devasenapathy et al, 2009  Measured |

Table S3. Effect of nano-urea on cost of cultivation, gross returns, net returns and B:C of maize cultivation

| Treatments | Cost of cultivation  (US $ ha^-1^) | | Gross returns  (US $ ha^-1^) | Net returns  (US $ ha^-1^) | | B:C | |
| --- | --- | --- | --- | --- | --- | --- | --- |
|  | With subsidized urea | With unsubsidized urea |  | With subsidized urea | With unsubsidized urea | With subsidized urea | With unsubsidized urea |
| N_0_PK | 507 | 507 | 834^D^ | 327^E^ | 327^E^ | 1.65^D^ | 1.65^D^ |
| N_50_PK | 533 | 599 | 1142^C^ | 609^D^ | 543^CD^ | 2.14^C^ | 1.91^BC^ |
| N_75_PK | 540 | 640 | 1329^B^ | 788^BC^ | 689^BC^ | 2.46^B^ | 2.08^AB^ |
| N_100_PK | 548 | 681 | 1506^A^ | 958^A^ | 825^AB^ | 2.75^A^ | 2.21^A^ |
| N_0_PK + Nano-urea | 538 | 538 | 957^D^ | 419^E^ | 419^DE^ | 1.78^D^ | 1.78^CD^ |
| N_50_PK+ Nano- urea | 563 | 630 | 1320^B^ | 757^C^ | 690^B^ | 2.34^BC^ | 2.10^AB^ |
| N_75_PK+ Nano- urea | 571 | 671 | 1482^A^ | 911^AB^ | 811^AB^ | 2.59^AB^ | 2.21^A^ |
| N_100_PK+ Nano- urea | 579 | 712 | 1623^A^ | 1044^A^ | 911^A^ | 2.80^A^ | 2.28^A^ |

*Values of means followed by different capital letter(s) (based on Duncan’s multiple range tests) within the row are significantly different at p ≤0.05.

***Note:*** Price for one bag subsidized urea (45 kg) was **US $** **3.36** and unsubsidized urea was **US $** **28.38** taken. 1US $ = ₹ 79.29 as on 09 July 2022.

Table S4. Effect of nano-urea on cost of cultivation, gross returns, net returns and B:C of wheat cultivation

| Treatments | Cost of cultivation  (US $ ha^-1^) | | Gross returns  (US $  ha^-1^) | Net returns  (US $ ha^-1^) | | B:C | |
| --- | --- | --- | --- | --- | --- | --- | --- |
|  | With subsidized urea | With unsubsidized urea |  | With subsidized urea | With unsubsidized urea | With subsidized urea | With unsubsidized urea |
| N_0_PK | 447 | 447 | 731^G^ | 284^E^ | 284^E^ | 1.63^D^ | 1.63^E^ |
| N_50_PK | 470 | 523 | 1019^E^ | 548^D^ | 495^D^ | 2.17^C^ | 1.95^CD^ |
| N_75_PK | 476 | 556 | 1219^CD^ | 743^BC^ | 663^BC^ | 2.56^B^ | 2.19^AB^ |
| N_100_PK | 483 | 589 | 1334^AB^ | 852^A^ | 745^AB^ | 2.77^A^ | 2.27^A^ |
| N_0_PK + Nano-urea | 478 | 478 | 856^F^ | 378^E^ | 378^E^ | 1.79^D^ | 1.79^DE^ |
| N_50_PK+ Nano- urea | 501 | 554 | 1158^D^ | 657^C^ | 604^C^ | 2.31^C^ | 2.09^BC^ |
| N_75_PK+ Nano- urea | 507 | 587 | 1294^BC^ | 787^AB^ | 707^AB^ | 2.55^B^ | 2.21^AB^ |
| N_100_PK+ Nano- urea | 514 | 620 | 1392^A^ | 878^A^ | 772^A^ | 2.71^AB^ | 2.25^AB^ |

*Values of means followed by different capital letter(s) (based on Duncan’s multiple range tests) within the row are significantly different at p ≤0.05.

***Note:*** Price for one bag subsidized urea (45 kg) was **US $** **3.36** and unsubsidized urea was **US $** **28.38** taken. 1US $ = ₹ 79.29 as on 09 July 2022.

Table S5. Effect of nano-urea on cost of cultivation, gross returns, net returns and B:C of pearl millet cultivation

| Treatments | Cost of cultivation  (US $ ha^-1^) | | Gross returns  (US $ ha^-1^) | Net returns  (US $ ha^-1^) | | B:C | |
| --- | --- | --- | --- | --- | --- | --- | --- |
|  | With subsidized urea | With unsubsidized urea |  | With subsidized urea | With unsubsidized urea | With subsidized urea | With unsubsidized urea |
| N_0_PK | 332 | 332 | 722^C^ | 391^C^ | 391^C^ | 2.18^E^ | 2.18^D^ |
| N_50_PK | 343 | 370 | 975^B^ | 632^B^ | 606^B^ | 2.85^D^ | 2.64^C^ |
| N_75_PK | 346 | 386 | 1073^B^ | 727^B^ | 687^B^ | 3.10^CD^ | 2.78^BC^ |
| N_100_PK | 349 | 402 | 1250^A^ | 901^A^ | 848^A^ | 3.58^A^ | 3.11^A^ |
| N_0_PK + Nano-urea | 362 | 362 | 834^C^ | 472^C^ | 472^C^ | 2.30^E^ | 2.30^D^ |
| N_50_PK+ Nano- urea | 374 | 400 | 1083^B^ | 709^B^ | 682^B^ | 2.90^D^ | 2.70^C^ |
| N_75_PK+ Nano- urea | 377 | 418 | 1219^A^ | 842^A^ | 802^A^ | 3.23^BC^ | 2.92^ABC^ |
| N_100_PK+ Nano- urea | 380 | 433 | 1309^A^ | 929^A^ | 876^A^ | 3.44^AB^ | 3.02^AB^ |

*Values of means followed by different capital letter(s) (based on Duncan’s multiple range tests) within the row are significantly different at p ≤0.05.

***Note:*** Price for one bag subsidized urea (45 kg) was **US $** **3.36** and unsubsidized urea was **US $** **28.38** taken. 1US $ = ₹ 79.29 as on 09 July 2022.

Table S6. Effect of nano-urea on cost of cultivation, gross returns, net returns and B:C of mustard cultivation

| Treatments | Cost of cultivation  (US $ ha^-1^) | | Gross returns  (US $  ha^-1^) | Net returns  (US $ ha^-1^) | | B:C | |
| --- | --- | --- | --- | --- | --- | --- | --- |
|  | With subsidized urea | With unsubsidized urea |  | With subsidized urea | With unsubsidized urea | With subsidized urea | With unsubsidized urea |
| N_0_PK | 346 | 346 | 877^E^ | 531^D^ | 531^D^ | 2.54^E^ | 2.54^D^ |
| N_50_PK | 360 | 395 | 1208^C^ | 848^C^ | 813^C^ | 3.36^D^ | 3.06^BC^ |
| N_75_PK | 364 | 417 | 1392^B^ | 1028^B^ | 975^B^ | 3.82^BC^ | 3.34^AB^ |
| N_100_PK | 368 | 439 | 1575^A^ | 1207^A^ | 1136^A^ | 4.28^A^ | 3.59^A^ |
| N_0_PK + Nano-urea | 377 | 377 | 1042^D^ | 665^D^ | 665^CD^ | 2.76^E^ | 2.76^CD^ |
| N_50_PK+ Nano- urea | 390 | 426 | 1399^B^ | 1009^B^ | 973^B^ | 3.58^CD^ | 3.29^AB^ |
| N_75_PK+ Nano- urea | 395 | 448 | 1524^AB^ | 1130^AB^ | 1077^AB^ | 3.86^BC^ | 3.40^AB^ |
| N_100_PK+ Nano- urea | 399 | 470 | 1639^A^ | 1240^A^ | 1169^A^ | 4.11^AB^ | 3.49^A^ |

*Values of means followed by different capital letter(s) (based on Duncan’s multiple range tests) within the row are significantly different at p ≤0.05.

***Note:*** Price for one bag subsidized urea (45 kg) was **US $** **3.36** and unsubsidized urea was **US $** **28.38** taken. 1US $ = ₹ 79.29 as on 09 July 2022.
