# Supplementary figures and images for: Conjoint application of nano-urea with conventional fertilizers: An energy efficient and environmentally robust approach for sustainable crop production
Source: PLoS One. 2023 Jul 5;18(7):e0284009. doi: 10.1371/journal.pone.0284009 (PMC10321634; doi:10.1371/journal.pone.0284009)

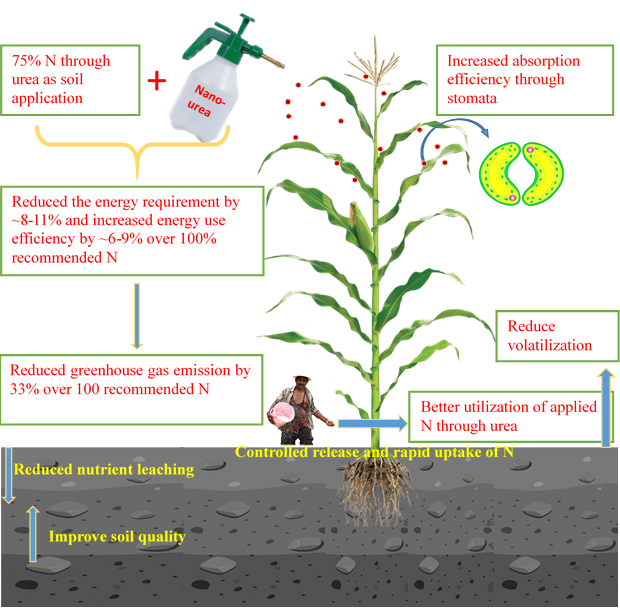

Supplement: S1 Graphical abstract — (TIF) [file pone.0284009.s002.tif]
